# Multi‐atlas fully automated segmentation of the locus coeruleus in in vivo neuromelanin‐sensitive MRI

**DOI:** 10.1002/alz.093783

**Published:** 2025-01-09

**Authors:** Robin de Flores, Thaïs Blanchard, Emilie Foyard, Mikaël Naveau, Nicolas Delcroix, Brigitte Landeau, Paul A. Yushkevich, Gael Chételat

**Affiliations:** ^1^ Normandie Univ, UNICAEN, INSERM, U1237, PhIND Physiopathology and Imaging of Neurological Disorders, NeuroPresage Team, GIP Cyceron, Caen France; ^2^ UMS3408 CYCERON, CNRS, Univ Caen Normandie, Caen France; ^3^ Perelman School of Medicine, University of Pennsylvania, Philadelphia, PA USA

## Abstract

**Background:**

Locus coeruleus (LC) imaging using neuromelanin‐sensitive (NM) MRI sequences is a promising biomarker for detecting early Alzheimer’s Disease (AD). Although automatic approaches have been developed to estimate LC integrity by measuring its intensity, these techniques most often rely on a single template built in a standardized space and/or depend on a number of voxels to be accounted that is defined a priori. Thus, these algorithms make it impossible to perform direct volumetric analyses and do not properly account for inter‐individual anatomical variability. To fill this gap, our aim was to develop a new multi‐atlas fully automated segmentation method using the Automatic Segmentation of Hippocampal Subfields (ASHS) software.

**Method:**

We used baseline data from 102 unimpaired older adults (mean age: 73.72 ± 3.5 years; mean education: 13.25 ± 3.1 years; 58 women, 44 men) from the Age‐Well randomized controlled trial for whom high‐resolution NM MRI (T1‐w with magnetization transfer; 0.3×0.3×0.75mm3) and standard T1‐w MRI (1×1×1mm3) were available. The LC were manually segmented in 30 randomly selected participants on NM MRI, and the standard T1‐w MRI, NM MRI and bilateral segmentations were fed into the ASHS training pipeline to generate an atlas. This new atlas was applied to the 72 remaining subjects to segment the LC and we assessed the effects of age, sex and education on both i) LC intensity (normalized by the intensity of the pons) and ii) LC volume (normalized by the total intracranial volume).

**Result:**

Five‐fold cross‐validation experiments revealed high accuracy of the automatic segmentation relative to manual segmentation (Dice coefficient = 0,83 ± 0,04). LC intensity was significantly higher in women than in men (F = 13.61,p<0.001) while no associations with age (ß = ‐0.0002,p = 0.86) or education (ß = 0.002,p = 0.16) were found. In contrast, LC volume was not different between men and women (F = 0.21, p = 0.65) but tended to be negatively associated with age (ß = ‐0.15,p = 0.06) and education (ß = ‐0.19,p = 0.06).

**Conclusion:**

Overall, this new method allows to automatically and accurately segment the LC, and offers the opportunity to measure its integrity both in terms of intensity and volume. This is of importance since these two metrics might provide complementary information about the integrity of the LC.

